# Indents

**Published:** 1917-11

**Authors:** 


### INDENTS

☞ **Brief Articles of Technical or Scientific Value will receive notice and comment. Contributions invited.**

*Ipecac in Treatment of Pyorrhea* — *The Journal of American Medical Association* for October 20 has this to say for this preparation: In spite of the enthusiastic advocacy, in the past, of ipecac alkaloids as a specific in pyorrhea alveolaris, the preponderance of scientific evidence indicates that ipecac is of questionable value in this condition. Neither is there any substantial evidence to warrant the claim that ipecac alkaloids, when absorbed through the intestines, are demonstrably useful in amebic infections of the tonsils.

*Necrobiosis the Cause of Pyorrhea* — There can be no primary infection without pain, tenderness or soreness. We absolutely know that many cases of pyorrhea never manifest these symptoms, and that no case is tender or sore in its early stages. If the germ theory be true, soreness would be manifested from the very first. We are then driven to the conclusion that here we have a destruction of tissue without infection which is necrobiosis. Often tenderness and soreness appear later when there *is* infection, but this is secondary, and results in the formation of pus as in all other infections.

What causes the death of the parts resulting in a so-called pyorrhea? To answer this question we must go to the very fountain of life, the heart. The failure of the heart to get the life-giving fluid to these parts. This being cut off, death must ensue.

In nearly all persons twenty-five years old and over we find pyorrhea. The reason for this is plain. The arteries are less vascular, and it is difficult for the heart to force the blood through these very small vessels which supply the thin bone holding the tooth. In young persons, while the arteries are soft, we find this trouble rarely, and in all cases in persons under twenty years, a weak heart action will be noticed.

In all the literature upon the subject of pyorrhea will be found the statement that it is caused by tartar, injury to the gums, overhanging fillings or malocclusion. As a matter of fact, none of these things produce this trouble. I have seen mouths where the only places not affected were those where there was an accumulation of tartar. It has been noticed by all observers that long, strong teeth, with no deposits whatever, are often badly affected with this disease. Often these mouths have had the best of attention and still the teeth begin to loosen and drop out. In other mouths, where all the above "causes" are present, we find no pyorrhea. Can this be explained by the germ theory? Not at all, but it can be explained by the necrobiosis theory.

The longer the tooth the longer the thin bone which is to be supplied with blood; hence the more difficult to supply with blood. In short teeth, deeply set in the alveolar process, the more easily can the heart and arteries supply the life-giving fluid.—E. P. Beadles in *Summary*.

*Gold or Aluminum Lining for Vulcanite Dentures*

When the wax baseplate has been removed from the mold, just before packing in the rubber, coat the cast with liquid silex and immediately sift on some powdered aluminum or gold, as you may desire. Now dissolve a piece of the same kind of rubber used for the plate in chloroform and add double the amount of powdered aluminum to the bulk of the solution and by means of a camel's brush coat the cast. Allow sufficient time for the chloroform to evaporate before packing and closing the flash. A lining prepared in this way is durable and can not pull off. The lining prevents any action of silex on the plate in vulcanizing and the silex prevents the plaster from sticking to the palatal portion of the plate.—T. W. Crozier, *Dental Digest*.

*The Danger of Mouth Spray Infection*

Under conditions that involve the close association of large numbers of persons, as in the great military encampments, certain modes of infection acquire a kind of exaggerated importance. The transmission of disease germs in ways that are rare in normal everyday life occurs with greater frequency and has more far-reaching consequences. Exposure to inhalation of mouth spray is one of the most serious dangers. The small droplets discharged from the mouth or nose in sneezing, coughing, laughing and loud talking float about in the air for some time, and may be carried by currents of air to a considerable distance. There is reason to believe from the experiments of Köhlisch with tubercle bacilli that the moist droplets which often contain a little mucus are more likely than dry dust to produce infection. Köhlisch, for instance, found in animal experiments that fifty tubercle bacilli inhaled as bacterial spray produced over twenty lung nodules, while the inhalation of over 2,000 in the form of dry dust had a very irregular action, and in some cases evoked no pathologic change. It is a somewhat general belief that, in human tuberculosis, infectious droplets are a more frequent source of infection than dried sputum. In numerous other infections that enter by way of the respiratory tract, the inhalation of mouth spray coming from an infected person or healthy carrier seems the typical mode of infection. This is true in diseases like pneumonia, influenza and whooping cough, and probably in measles. Epidemic meningitis is probably also most commonly spread in this way. The importance of mouth spray in surgical wound infection is well known. In camp life, infection of this sort is notably difficult to control. The rapidity with which measles and epidemic meningitis are likely to spread among new recruits is typical. Possibly increase of tuberculosis among troops is partly due to the increased opportunity for infection as well as to the influence of predisposing factors. It is doubtful whether hygienic instruction in the use of the handkerchief for smothering mouth spray from the particularly mischievous sneezes and coughs would be immediately effective, although every hygienically trained individual is a help toward the desired end. General camp measures that promote resistance to infection are always of value, probably especially so in meningitis, less of course in measles. If it were possible in cases of "cold" and the like in the incipient stage to make examinations, and, when necessary, to isolate the patients, the spread of mouth spray infection might be materially checked. This measure is worthy of consideration in all conditions in which there is any definite and serious respiratory tract epidemic. Known contacts may be kept under special surveillance and the cooperation of officers enlisted to detect early signs of infection. There is no single and universally applicable method for preventing the transference of germs from one soldier's respiratory tract to another's, but all reasonable and persistent attempts to prevent mouth spray infection are worth making. A person with the developed rash of measles or the skin-peeling of scarlet fever is not allowed to roam unchecked, if his condition is known; but little or no restraint is yet put either by health authorities or by public opinion on the wanderings of the much more dangerous person who is sneezing and coughing myriads of germs into the atmosphere.—*Journel A. M. A.*, October 13.

*Food Values* Mr. Hoover's instruction card and the government devices are variations of lessons in food values. Every one understands perfectly that four quarters may take the place of one dollar in buying food, but many people can not tell how many eggs at 40 cents a dozen may be used to replace in food value round steak at 25 cents a pound, or how to substitute for a quart of milk. It is easy even now to see that the term "food value" is beginning to receive respectful attention, and that way lies the basis for wise buying of food. War tends to make kindred of men. We can forgive the erstwhile scoffer, and we urge him to satisfy his newly acquired curiosity as to food values by consulting a timely series of well-edited bulletins on "How to Select Foods," which the United States Department of Agriculture has lately put at the disposal of the public without cost.

These are issued under the editorship of Caroline L. Hunt and Helen W. Atwater as Farmers' Bulletins. They are available for free distribution on postal card request to the Department of Agriculture at Washington.—*Journal A. M. A.*

*Radiographing Root Canals* I get better results by the use of iridio-platinum wires in root canals that I radiograph. Keep an assortment of different sizes and lengths and finish to a fine point at one end. I heat them to sterilize before and after using them and use the same one indefinitely. Gauges 23, 24 and 25 are the sizes I use mostly. You will find that this saves time, having them ready and tends to standardize the work.—Homer Almon, *Dental Review.*

*Applying Devitalizing Paste* Remove enough of the decay and thin edges of the cavity to get acess to the pulp; select a suitable shaped instrument and place on its point nerve paste of the consistency of thick cream and about the bulk of one-fourth of a common pin head; touch this to some muriate of cocaine crystals, enough of which will adhere to benumb the nerve in most cases; place with a sliding motion of the instrument to free it from the application. If in a lower tooth, flow over it thinly mixed calxine. If an upper tooth, moisten (but do not saturate) a pellet of cotton with calxine liquid. Mix calxine liquid and powder thin, saturate the moistened cotton and place without pressure in contact with the nerve paste. Finish filling the cavity with calxine mixed a little thicker. Have the patient return in one week or earlier in case the tooth begins to get sore.—D. S. Thomas, in *Oral Hygiene.*

*Treating Root Perforations* Allow me to state an easy method of treating and repairing a perforation of the pulp chamber or the canals, as this will occur in the best of regulated practices: A sedative dressing of equal parts of oil of cloves and beechwood creosote should be placed over the perforation and allowed to remain until the canal can be dried without hemorrhage; the perforation should be varnished with a solution of rosin in cholorform, and a piece of warm guttapercha should then be placed over the opening and pressed to place with a warm plugger. If the perforation is near the apical foramen, a guttapercha point will usually obliterate it. These operations should be checked by an X-ray picture.—F. C. Pearn, *Northwestern Journal of Dentistry.*

*The Menace of Diseased Teeth* These suppurative detachments of the peridental membrane are in practically all cases permanent detachments, whether the detachment is at the side of the root or the apex. The area of bone destroyed about the apex of a root is not so important as the extent of the destruction of the peridental membrane. There is no hope of reattachment of the surrounding tissue to the root, and if such teeth are permitted to remain in the mouth—excepting those which are operated on by resection—it should be with the definite understanding that they necessarily continue as a menace to the health of the individual, and that the use of such teeth in mastication overbalances this menace to the health. In such cases we are using our best judgment as to the patient's general physical condition and his resistance. We must do this with the thought ever in mind that nephritis, endocarditis, cholecystitis and other secondary effects are so insidious in their onset that the condition is likely to be serious and the patient ever beyond the possibility of recovery before it is discovered by the physician.—A. D. Black, in *Journal A. M. A.*, August 25, 1917.

*Explorer Pickings*

A dentist is known by the patients he keeps.

A loose tooth gathers lots of tartar.

Did you ever notice how uncomfortable every tooth is before the bill is paid?

A patient in nine pays all the time.

He who is drilled and runs away will live to have a heluvan ache some day.

Be sure and make that tooth safe for democracy but don't crown it.

A few articles of diet which all dentists ought to recommend: Corn on the cob, celery, crusts of bread, cordovan steak and hickory nut shells.

Would the power the gift to give us to see ourselves as our patients see us.—Earem, in *New Jersey Journal.*

*A Hint on Partial Lower Dentures*

Partial lower dentures supplying some or all of the bicuspids and molars sooner or later, on account of the absorption of the alveolar process, settle to such extent that the teeth of the denture do not reach up to and occlude with the teeth of the upper set.

Any attachments used for retaining the lower partial denture should be so constructed that they will adapt themselves to such changes as are caused by the absorption of the alveolar process.

The following method for raising the lower partial denture so that the teeth of the denture will properly occlude with the upper set will be found to be practical and easy of accomplishment.

Drill numerous holes into those parts of the denture that rest upon the gum, then place one or more sheets of wax over those surfaces, according to the amount of absorption that has taken place, then warm the wax in hot water to soften it, and place in the mouth and tell the patient to bite, bringing the teeth of the denture into proper occlusion with the upper set. Then cool the wax while in the mouth, and remove and trim off the surplus wax, then flask the case upside down and vulcanize rubber in place of the wax which will restore the denture to its original position in the mouth.—H. A. Cross, in *Review*.

*Make a Survey of the Mouth*

Before you begin to operate make a good general survey of the whole mouth by lifting up and out the lips and cheeks so you can get a clean view of the entire oral cavity. As a rule the patient comes simply because of a troubling tooth, but in reality the whole masticatory apparatus needs reconstructing, so don't begin to operate on the troubling tooth until you see, and have made up your mind as to what the whole mouth really needs. By so doing you will not only benefit the patient, but you will also enhance the value of your services in the eyes of your patient.—W. O. Fellman, in *Review*.

*To Reduce the High Cost of Bridge Work*

After backings have been adapted to Richmond facings, cut off the projecting pins, flush with the backing; split the remaining stump longitudinally with a sharp knife. This minimizes the danger of checked facings and saves 8½ cents per tooth in platinum.—P. G. Puterbaugh, in *Review*.

*Pulp Canal Treatment Errors*

1. Lack of appreciation of the importance of pulp conservation. (Pulp should always be saved, if possible.)

2. Lack of appreciation of the many dangers incident to and following the removal of the pulp.

3. Lack of appreciation of the necessity of surgical cleanliness in every stage of pulp treatment. This means practically the same careful treatment for dead pulps, infected pulps, abscessed conditions, etc., as for a normal, healthy pulp.

4. The dangers of injury to the involving tissues incident to the use of strong drugs.

5. Failure to make fillings during the treatment period that hermetically seal. (Lack of thorough curettement.)

6. Failure of the dentist to receive a fee worthy of this most important operation.

Can the profession be brought to realize that the pulp tissue within the tooth is not different from other tissues of the body as far as carrying infection is concerned? Can we be brought to realize that all procedures connected with pulp treatment should be performed with surgical cleanliness? Does any well-informed dentist believe that even 50 per cent. of the teeth under treatment today are being treated by a surgically clean method? One pertinent question is sufficient to cause us to think twice on this last question.

When the average operator places cotton which he has wound on a broach into a canal, is it a sterile piece of cotton? Where did he get the cotton and how did he wind it on the broach? The great percentage of operators will take cotton from an open cotton receptacle that stands on the tray week in and week out. This same cotton is the supply base for almost all operations. It is usually wound between fingers that are anything but sterile. I could continue at length on these and similar points, but I know it is not necessary. The point that I want to make right here is this: Get away from the idea that you are only removing a nerve. Get your patients away from the idea that you are only removing a nerve. Educate them to know that the proper removal of a pulp is a surgical procedure and is subject to the same laws, the same dangers that surgical interference in the other tissues involve, namely, blood poisoning. If patients only knew the dangers they are continually being exposed to, do you believe they would permit these careless operations?

The present tendency among the leading surgeons of the day, with the exception of the surgery on the European battlefields, is to discard the use of antiseptics of any kind within the tissue. This is a complete reversal of the practice of many years. It is true that the use of antiseptics inhibits bacterial growth, but it also inhibits nature from making a normal repair. The present tendencies of many of the dental profession is to continue in the use of strong medicine within the tissues. I give way to no man in my zeal for strict surgical cleanliness in every detail pertaining to root canal treatment, and I realize that the agents frequently used will very quickly destroy bacteria, but many times they do exactly what we do not want them to do—they inhibit the normal repair of the investing tissue or they destroy it. A rule that I should always apply would be this: Never use anything in the treatment of a root canal that can possibly injure the delicate tissues at the end of the root. It is unnecessary—it is unwise—it is dangerous.

I do not use arsenic for pulp destruction because I am convinced that many times the arsenic is responsible for the injury to the investing tissue. I am satisfied that arsenic in the hands of careful and skillful men may be used under certain prescribed conditions without much danger, yet I recognize that it is a dangerous drug, and I do not use it because it is dangerous and because I want to recommend its disuse. A loaded revolver may be safely used by some men, but when it will serve the same purpose in less skillful hands without being loaded, why run the chance? There are so many other methods of pulp removal that it is quite unnecessary to use this powerful drug.—F. W. Gethro, in *Review.*

*Dental Operations in Canadian Army*

From July 15, 1915, to June 30, 1917, the Canadian army dentists have made 955,335 operations of various kinds: Fillings, 384,741; treatments, 111,301; dentures, 74,572; prophylaxis, 43,565; extractions, 297,697; pulps devitalized, 43,459. This certainly makes good the claim of need for dental services in the army. It is earnestly hoped that our soldiers may have as good or even better service, in spite of the fact that we shall have only one dentist for every one thousand soldiers.

*An Act Providing for the Education and Examination of Dental Hygienists in Connecticut*

Be it enacted by the Senate and House of Representatives in General Assembly convened:

Section twelve of chapter 316 of the public acts of 1915 is amended to read as follows: Any registered or licensed dentist may employ women assistants, who shall be known as dental hygienists. Such dental hygienists may remove calcareous deposits, accretions and stains from the exposed surfaces of the teeth and directly beneath the free margins of the gums, but shall not perform any other operation on the teeth or mouth or on any diseased tissues of the mouth. They may operate in the office of any registered or licensed dentist or in any public or private institution under the general supervision of a registered or licensed dentist. The dental commission may revoke the license of any registered or licensed dentist who shall permit any dental hygienist operating under his supervision to perform any operation other than that permitted under the provisions of this section. On or after July 1, 1917, no dental hygienist shall be permitted to practice who has not registered with the recorder of the dental commission, unless such person shall pass an examination prescribed by the dental commission. The fee for such examination shall be ten dollars. Any applicant failing to pass such examination shall be entitled to a re-examination at the next meeting of the commissioners, without additional cost, and for any other additional examination a fee of five dollars shall be paid. The dental commission shall make such rules and regulations as may be necessary for the examination of dental hygienists. Said commission may issue its certificate to any applicant therefor who shall furnish proof satisfactory to said commission that she has been duly licensed to practice as a dental hygienist in another state after full compliance with the requirements of its dental laws, provided her professional education shall not be less than that required in this state. The dental commission may revoke the registration and license of any dental hygienist violating any provision of this act.

*Ethical: What Does It Mean?* Those of us who do not jar the professional code of ethics by displaying our names and occupations upon billboards are certainly deceiving ourselves into the belief that we are thereby ethical dentists. But we are not, if in addition to that our aims are not constructive. The truly ethical man is not the one who does not advertise and stops there. He is an ethical man who places the welfare of his fellowmen above all mercenary considerations and labors to the end that the greatest good may be showered upon the greatest number, regardless of creed and color and as often as opportunity renders it possible. He is ethical who spreads broadcast the gospel of good health, of moral standards, of cheerfulness, of love and kindness to young and old alike that it may all rebound to the greater efficiency and happiness of the human race.

*Removing Broken Pin from Detached Post Crown* To remove the broken pin from a Davis or other detached post crowns, immerse the crown in dilute sulphuric acid for a few hours, when the pin can be easily removed and the crown used again, with a pin of proper length.—*Pacific Gazette.*

*To Secure Exact Occlusion* For full sets of teeth, it is often difficult to get the occlusal exact and "flat." Some prosthetists take emery flour and mix with oil. After remounting on the anatomical occluding frame they smear the grinding surfaces with this, then work the sets and gradually wear the occluding surfaces until they close "flat." This is very tedious. Avoid this by smearing upon these surfaces hydrofluoric acid, being careful to use fresh acid, and only a little. Set the case aside for two or three hours. Now smear with emery powder and oil and the surfaces will easily wear down to that very desirable solid and flat occlusion. This also removes the glaze. The teeth will not dance or tip, other things being equal.—E. J. Perry, *Dental Review.*

*The Filling of Root Canals* Over fifteen years ago we had an occasion to call the attention of the profession to the desirability of avoiding any irritation of the tissues beyond the end of the root canal by either drugs or instrumentation, during the operation of removing the pulp or filling the root canal. In fact, we made the statement then that it might be better to leave a small portion of the pulp at the apex rather than to go through the end with irritating drugs, broaches, or root canal fillings. This teaching has been verified in the work of Carl G. Grove, Minneapolis, who states that there are fewer rarified areas around the apices of the roots of teeth not filled to the end than around those which are filled to the end by the methods above described. We can see no reason for saying that drug or even mechanical irritation around the end of the root may be the sole cause of reinfection, because it has not yet been shown that guttapercha will hermetically seal the end of a root; while, on the other hand, it has been shown by many experiments that guttapercha will not act as a barrier to bacteria.—Editorial, *Dominion Dental Journal.*

